# Sorption of Cellulases in Biofilm Enhances Cellulose Degradation by *Bacillus subtilis*

**DOI:** 10.3390/microorganisms10081505

**Published:** 2022-07-26

**Authors:** Yijie Deng, Shiao Y. Wang

**Affiliations:** School of Biological, Environmental and Earth Sciences, The University of Southern Mississippi, Hattiesburg, MS 39406, USA

**Keywords:** biofilm matrix, cellulose degradation, extracellular polysaccharides, *Bacillus subtilis*, cellulase, synergistic degradation, biofuel

## Abstract

Biofilm commonly forms on the surfaces of cellulosic biomass but its roles in cellulose degradation remain largely unexplored. We used *Bacillus subtilis* to study possible mechanisms and the contributions of two major biofilm components, extracellular polysaccharides (EPS) and TasA protein, to submerged biofilm formation on cellulose and its degradation. We found that biofilm produced by *B. subtilis* is able to absorb exogenous cellulase added to the culture medium and also retain self-produced cellulase within the biofilm matrix. The bacteria that produced more biofilm degraded more cellulose compared to strains that produced less biofilm. Knockout strains that lacked both EPS and TasA formed a smaller amount of submerged biofilm on cellulose than the wild-type strain and also degraded less cellulose. Imaging of biofilm on cellulose suggests that bacteria, cellulose, and cellulases form cellulolytic biofilm complexes that facilitate synergistic cellulose degradation. This study brings additional insight into the important functions of biofilm in cellulose degradation and could potentiate the development of biofilm-based technology to enhance biomass degradation for biofuel production.

## 1. Introduction

Biofilm is normally defined as a single-species population or multi-species microbial community embedded in a structured extracellular matrix attached to surfaces; the matrix consists mainly of polysaccharides, proteins, lipids, and extracellular DNA [1,2]. The extracellular matrix in biofilm performs functions critical to bacteria such as retention of bacterial cells on solid surfaces, immobilization of enzymes, protection of bacteria from hostile environments, and absorption and concentration of nutrients [1,2,3]. Beyond its importance in bacterial virulence in medicine, biofilm also plays an essential role in removing organic matter and the cycling of most nutrients in the environment and thus can be engineered for biotechnological applications including bioremediation and bio-production [1,2,3,4,5].

Biofilm is commonly found associated with cellulosic substrates during its slow degradation by adherent bacteria [6,7,8,9,10]. These cellulosic materials, mainly from plant biomass, provide carbon sources and surfaces for bacteria to grow and develop biofilm under moist or submerged conditions [11,12,13]. However, the function of biofilm in cellulose degradation and its underlying mechanism remains poorly studied. Given the insolubility of cellulose in water, we speculated that cellulose degradation relies on the cellulolytic bacteria that form biofilm on the substrate. However, it remains to be documented whether biofilm and its specific components could aid cellulose degradation. A good understanding of specific functions of biofilm components is also required for potential developments of biofilm-related technologies as an efficient way for cellulose degradation [14] because the bottleneck for current biofuel production is the lack of an efficient way to degrade the recalcitrant cellulose, which makes biofuel production cost-prohibitive [7,8,9,14].

In this work, we used *Bacillus subtilis* as a model because its biofilm formation is well characterized. The bacterium is highly abundant in the environment and is known to play important roles in the decomposition of complex organic matter [15,16]. *B. subtilis* can form macro-colony biofilm on moist surfaces, pellicle biofilm at the liquid-air interface, as well as a submerged biofilm on objects in liquid [17,18,19]. Extracellular polysaccharides (EPS) and TasA protein are two major components in *B. subtilis* biofilm [20,21], though some other components might also be involved including extracellular DNA and BslA protein [22,23]. The EPS are encoded by a 15-gene operon named *epsA-O*; deletion of this operon abolishes pellicle biofilm formation [21,24,25] and deletion of a single gene *epsG* in the operon prevents the formation of surface-adhered biofilm submerged in liquid [26]. Similarly, deletion of the *tasA* gene leads to defective pellicle biofilm formation [21]. Notably, the two major biofilm components, EPS and TasA, can assemble complementarily and extracellularly to form intact biofilm [21].

Taking advantage of this well-characterized bacterium, we investigated whether biofilm can capture and retain cellulases and promote cellulose degradation. We compared cellulase capture and cellulose degradation between the biofilm-knockout strains and the wild-type strain. Because the *B. subtilis* strains used in this study produced little cellulase, we first added cellulases to the growth medium and compared the capture of cellulase and the rate of cellulose degradation between the biofilm-deficient and wild-type strains. In a second study to ascertain the absorption of self-produced cellulase and its effect on cellulose degradation, we used the same bacterial strains but transformed with a plasmid containing a secretory cellulase gene. The objectives of the study were to determine (1) whether biofilm can capture cellulases, (2) whether enzyme localization by biofilm facilitates cellulose degradation, and (3) how biofilm matrix components, EPS and TasA, contribute to cellulose degradation.

## 2. Materials and Methods

### 2.1. Culture Media

A biofilm formation (BF) medium was modified from the medium used in a previous study [17]. Each liter of BF medium contained 10 g (NH_4_)_2_SO_4_, 5 g K_2_HPO_4_, 5 g KH_2_PO_4_, 5 g sodium citrate, 1 g glucose, 0.12 g of MgSO_4_, 5 g NaCl, 5 g tryptone, and 5 g yeast extract. The pH of the medium was adjusted to pH 6.7. BF medium allows *B. subtilis* to form both pellicle biofilm at the air-liquid interface and submerged biofilm in liquid. When needed, all culture media and Luria–Bertani (LB) agar plates used were supplemented with appropriate antibiotics at concentrations: erythromycin (1 µg/mL), spectinomycin (100 µg/mL), zeocin (20 µg/mL), or chloramphenicol (5 µg/mL).

### 2.2. Bacterial Strains and Construction of Biofilm Knockout Mutants

*B. subtilis* strains and plasmids used in this study are listed in Table 1. All the biofilm-deficient mutants used in this study were in-frame knockout mutants. The in-frame replacement method has little polar effects on downstream genes [27] and was used to create similar Δ*tasA* and Δ*eps* mutants in previous studies [21,28]. The mutant Δ*tasA* was directly obtained from the Bacillus Genetic Stock Center (BGSC), Ohio State University. The Δ*eps* is an in-frame knockout strain in which the *eps* operon was deleted using a long homologous flanking (LHF)-PCR technique [20,29] with a knockout cassette containing a zeocin selective marker. The upstream and downstream homologous fragments were PCR-amplified from *B. subtilis* 168 genomic DNA using primers eps-up-F/eps-up-R and eps-dw-F/eps-dw-R (Table 2). The zeocin-resistant gene (*zeoR*) was amplified from the plasmid p7Z6 [30] using primers zeo-F and zeo-R. The *eps* knockout cassette consisting of three fragments (eps-up, ZeoR, and eps-down) was made by joining PCR. The purified PCR product was used to transform *B. subtilis* following the classic method based on the natural competence of the bacterium [31]. Knockouts were selected on zeocin plates and deletion of the *eps* operon was verified by PCR. The double deletion knockout strain (Δ*tasA* Δ*eps*) was made by transforming the Δ*tasA* strain with the *eps* knockout cassette and selecting for zeocin resistant colonies. All knockouts were confirmed by PCR with appropriate gene-specific primers (Table 2) and phenotypic checking. Deletion of *tasA* was verified using primers tasA-F/tasA-R targeting *tasA* with an expected product size of ~500 bp. Deletion of the *eps* operon was verified using primers epsCF-F/epsCF-R with an expected product size of ~3.5 kb. Deletion of the *eps* operon and replacement by *zeoR* was verified using primers eps-up-F/Zeo-R with an expected product size of ~1.5 kb.

### 2.3. Estimation of Submerged Biofilm Formation on Cellulose

Submerged biofilm formation by all *B. subtilis* strains was compared using cellulose filter paper discs as substrate. Bacteria were grown in 12-well plates. Each well received 1 mL of BF medium with an initial bacterial density of OD_600_ = 0.02. A filter paper disc (13 mm diameter) was placed into each well as the substrate for biofilm formation. Bacteria in the 12-well plates were incubated on a rocking shaker (Reliable Scientific Inc., Hernando, MS, USA) to create back-and-forth flow and gentle agitation at 30 °C for 28 h before biofilm quantification. For the blank control, filter paper discs were incubated in the BF medium without bacteria.

To estimate the amount of submerged biofilm on filter paper discs, we developed a staining method based on earlier methods that used hematoxylin-eosin to detect biofilm on human tissues [34,35]. Eosin Y stains biofilm but not cellulose filter paper. To estimate the amount of biofilm, each filter paper disc was transferred to 2 mL of eosin staining solution (0.25 g eosin Y in 100 mL 80% ethyl alcohol with 0.5 mL acetic acid) in 12-well plates and stained for 15 min. Unbound dye was removed afterward by washing each stained filter paper disc individually in 120 mL distilled water twice for 20 min each. Each washed filter paper disc was then transferred to a 1.5-mL centrifuge tube in which biofilm-bound eosin dye was extracted in 1 mL DMSO. After centrifugation at 10,000× *g* for 2 min, the absorbance (540 nm) of the supernatant (150 µL) was measured using a microplate reader in a 96-well plate. The absorbance of blank controls was negligible and was subtracted from each sample.

### 2.4. Measurement of Cellulase in Biofilm and Supernatant

Because the *B. subtilis* wild-type and knockout strains produce little cellulase, a fungal cellulase (Worthington Biochem. Corp., Cat# LS002598, Lakewood, NJ, USA) was added to the growth medium in one study. Overnight cultures of the wild-type and knockout strains were diluted to OD_600_ = 0.6 (~2 × 10^8^ CFU/mL) with BF medium. The diluted bacterial suspensions (3 mL each) were transferred to 100 mm × 16 mm polypropylene tubes and cellulase was added to a final concentration of 53 µg/mL. The cultures were incubated at 30 °C for 24 h without shaking to allow for biofilm formation and cellulase absorption. Afterward, samples were centrifuged at 4000× *g* for 3 min, and the cellulase assay described below was performed on both the pellet containing biofilm and supernatant. Each biofilm pellet with cellulase absorbed was suspended in 0.4 mL of PBS buffer and the OD_600_ was measured to estimate the amount of biofilm before the cellulase assay. For a control to measure the amount of cellulase that adhered to the cell surface, 3 mL of the wild type grown overnight with vigorous shaking to inhibit biofilm formation (diluted to OD_600_ = 1.0 in BF medium) was incubated with the same amount of fungal cellulase for 2 h and the amount of cellulase in cell pellets measured. Binding at the molecular level happens rapidly, normally between a few minutes to a couple of hours [36,37] and we assumed that binding of cellulase to cell surfaces would reach the equilibrium in less than two hours.

Cellulase activity was measured using a modified microplate-based dinitrosalicylic acid (DNS) method where cellulase breaks down the substrate carboxymethyl cellulose (CMC) into glucose which can be measured with DNS reagent [38]. Briefly, 40 µL of sample and 40 µL of 2% CMC solution were combined in wells of a 96-well PCR plate and incubated at 50 °C for 30 min. To determine the amount of released glucose, DNS reagent (80 µL) was added to each well, followed by incubation at 95°C for 5 min for color development. Each sample (120 µL) was transferred to a new 96-well microplate and the absorbance was measured at 540 nm using a Synergy 2 microplate reader (Bio-Tek Instruments Inc., Winooski, VT, USA). A standard curve was constructed for each experiment using glucose. Cellulase level was calculated based on the amount of glucose present and expressed in international units with one unit defined as one µmol of glucose released per min [38].

Note that cellulase sorption in biofilm was measured in two studies, one using wild-type and knockout strains that produce little cellulase and another using the same strains transformed with a plasmid to enhance self-produced cellulase (described below). In the former, the same amount of cellulase was added to all cultures and the unit of measure for cellulase sorption is U/OD (enzyme activity per unit biofilm). In the latter, to adjust for variability in cellulase production among transformed stains, the unit of measure is the percentage cellulase in biofilm relative to total cellulase produced (described below).

### 2.5. Pretreatment of Cellulose and Filter Paper for Efficient Degradation

Cellulose filter paper (Whatman, Qualitative, Grade 1, Cole-Palmer, Vernon Hills, IL, USA) and microcrystalline cellulose powder (Sigma-Aldrich, cat# 310697, St. Louis, MO, USA) were pretreated with concentrated phosphoric acid [39,40] before being used as substrates for biofilm formation and cellulose degradation. This pretreatment resulted in digestible filter paper discs that were consistent in mass when dried and weighed. The cellulose powder was pretreated to make regenerated amorphous cellulose (RAC) as described previously [40] and quantified by the phenol-sulfuric acid method [41]. Filter paper discs (25 mm diameter) were dried overnight at 60 °C before being added to 15 mL of 83% phosphoric acid in Petri dishes (90 mm diameter) for 12 h. Each Petri dish contained 10 filter paper discs. The filter paper discs were then transferred to a glass beaker with running distilled water. After washing with running distilled water for 24 h to remove phosphoric acid, NaOH solution (1 M) was added to the beaker to adjust the pH to 6.5. The prepared RAC and filter paper discs were used as a substrate to study biofilm formation and cellulose degradation.

### 2.6. Degradation of Regenerated Amorphous Cellulose by Biofilm and Enzyme Complexes

Overnight cultures of the wild-type and the double knockout strains were diluted to OD_600_ = 0.6 before growth in BF medium in 12-well plates. Each well contained 2 mL of medium with 0.9% RAC as the substrate for biofilm formation and cellulose degradation. Bacteria were incubated on a rocking shaker (18 motions per minute) at 30 °C. After incubation for 8 h, 20 µL of 8 mg/mL fungal cellulase was added to each well. For bacteria-alone control wells, 20 µL of PBS buffer instead of cellulase was added. For cellulase-alone control wells, culture medium and cellulase were added but not bacteria. Blank control wells contained 2 mL of culture medium and 20 µL of PBS buffer. Each treatment had six replicates. The bacterial cultures and all the controls were incubated in 12-well plates for another 2 days. The RAC remaining after the incubation period was washed and then quantified using the phenol-sulfuric acid method [41] using glucose as the standard. As cellulose is a polymer of anhydroglucose, a conversion factor of 162/180 was applied to calculate the amount of cellulose remaining in each well.

### 2.7. Visualization of Biofilm on Cellulose

To visualize biofilm on cellulose, the wild-type strain was labeled by green fluorescent protein (GFP) using the integration vector pDR111-GFP(Sp) [33] at the *amyE* locus of *B. subtilis* that does not affect biofilm formation and growth [18]. After growth, the remaining cellulose together with bacterial biofilm was stained with a Congo red solution (final concentration 0.01%) for 15 min before examination by confocal laser scanning microscope (CLSM, Zeiss 510 META). A drop of the stained RAC with biofilm growth was gently transferred to a glass slide and a cover slip was applied. The slide was scanned using a 20× objective lens at an excitation wavelength of 488 nm. The emitted fluorescence was examined at 510 nm for GFP and 650 nm for Congo-red-stained cellulose.

### 2.8. Engineering Bacteria to Produce a Secretory Cellulase

The *B. subtilis* strain 168 is not efficient in degrading crystalline cellulose due to the low expression level and low activity of its indigenous cellulase BsCel 5 (EglS, GenBank: CAA82317) [42]. To confirm that biofilm can trap cellulase produced by bacteria living within and facilitate cellulose degradation, the wild-type and knockout strains were transformed with a mutated recombinant BsCel5 cellulase, MT2C, previously shown to increase the degrading efficiency of insoluble cellulose by *B. subtilis* [40,42]. The recombinant secretory BsCel5 cassette consists of a strong and constitutive promoter P43, an optimized ribosomal binding site (RBS), a signal peptide of *nprB* gene, and the BsCel5 gene that together enhance cellulase expression and secretion [40,42,43]. The genetic codons of *npr*B and BsCel5 were optimized to improve the expression level [44] using the IDT codon optimization tool at https://www.idtdna.com/CodonOpt, accessed on 15 July 2014. In addition, we designed a synthetic RBS sequence using RBS calculator [45] to further increase the level of cellulase expression [46,47]. The recombinant BsCel5 cassette including restriction sites was commercially synthesized (GENEWIZ Inc., South Plainfield, NJ, USA) and cloned into the high-copy-number plasmid pGVD1 containing a chloramphenicol resistance gene [32] via restriction sites SacI and BamHI, yielding a cellulase over-expression plasmid pBC. This plasmid was introduced to all strains and tested for cellulase activity. Each strain (3 mL with initial OD_600_ = 0.03) was grown under vigorous shaking conditions (200 rpm) for 30 h at 30 °C in BF medium with chloramphenicol (5 µg/mL). The final cell density (OD) and cellulase produced were measured as described above.

Cellulase production under the biofilm-forming condition was determined by measuring cellulase retained within the biofilm and in the supernatant. Briefly, the wild type and the double knockout mutant were grown in 12-well plates with the same initial density (OD_600_ = 0.3) for 2 days on a rocking shaker with gentle agitation (3 rocking motions per minute), allowing biofilm to form. The samples were then centrifuged at 5000× *g* for 3 min. Cellulase in the supernatant and the pellets, both resuspended in 0.4 mL PBS buffer, were measured as described above. The percentage of cellulase in the biofilm pellet relative to total cellulase was calculated. All data were normalized to the same cell density (OD 1.0) to correct for differences among cultures in biomass.

### 2.9. Filter Paper Degradation by Engineered Bacteria

Overnight cultures were grown in 12-well plates, each well containing 2-mL diluted culture (OD 0.02) in BF medium with chloramphenicol (5 µg/mL) and one pretreated 25 mm filter paper disc. The 12-well plates were incubated on a rocking shaker with 18 rocking motions per minute at 30 °C for 4 days. Afterward, the amount of cellulose digested was measured based on the loss in mass of filter paper discs. Blank medium with pretreated filter paper discs served as the negative control. The amount of cellulose in each filter paper disc was determined gravimetrically as described previously [48]. Briefly, filter paper discs were washed in a nitric acid/acetic acid reagent (prepared by mixing 150 mL 80% acetic acid and 15 mL concentrated nitric acid) [49] in boiling water for 30 min to remove all attached bacterial cells and biofilm, dried to constant weight at 60 °C and then weighed (to the nearest 0.1 mg). Each treatment had at least six replicates. Bacteria on filter paper discs were enumerated by colony-forming unit (CFU) on LB plate with appropriate antibiotics. The pretreated filter paper discs were transferred to PBS buffer after a brief rinse and attached bacteria were dislodged and suspended in the buffer by vortex with 2-mm zirconia beads (Laboratory Supply Network, Inc., Atkinson, NH, USA) before serial dilution and plating on LB plates.

### 2.10. Statistical Analysis

The statistical differences among groups were first analyzed by one-way ANOVA. Two-tail Student’s T tests were then used to compare the difference between the two groups. Significant differences between two groups were indicated by asterisks, with * for *p* < 0.05, ** for *p* < 0.01 and *** for *p* < 0.001. All analyses were performed in Microsoft Excel and/or RStudio (https://www.rstudio.com/, accessed on 10 January 2016).

## 3. Results

### 3.1. Differential Biofilm Formation on Cellulose Discs

**Figure 1:**
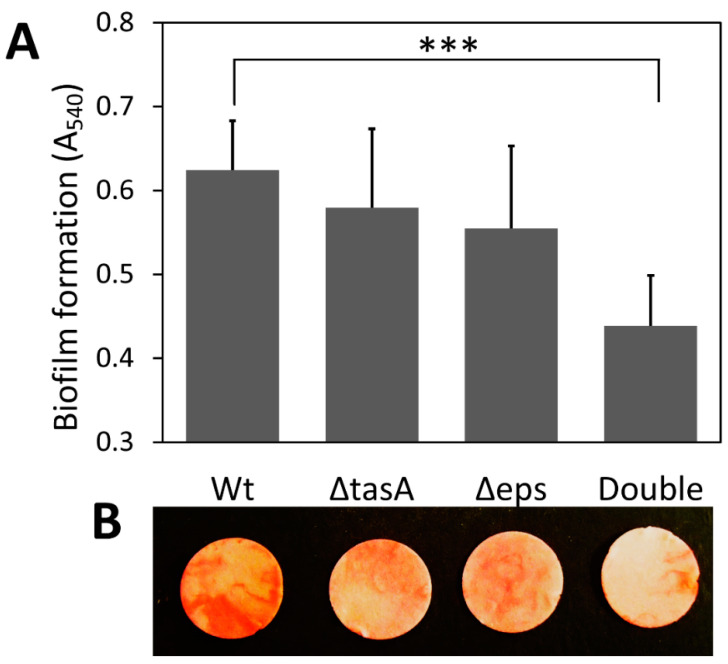
Effect of missing matrix components on submerged biofilm formation on cellulose discs. (**A**) Amount of biofilm on filter paper discs as determined by eosin Y staining. Values are means with one standard deviation (*n* = 6). (**B**) Images of biofilm on cellulose discs stained with eosin Y. The images were captured using a digital camera (Nikon Coolpix AW120). Wt: wild-type strain; Δ*tasA*: *tasA* knockout strain; Δ*eps*: *eps* knockout strain; Double: double-knockout (Δ*eps* Δ*tasA*) strain. Statistic differences between groups were analyzed by Student’s T tests, with *** *p* < 0.001.

Deletion of either or both genes that encode two major components of the biofilm matrix, extracellular polysaccharides (EPS) and TasA protein, did not affect cell growth but reduced biofilm formation (Figure 1 and Figure S1). The successful construction of all biofilm knockout strains was confirmed by PCR with gene-specific primers and phenotypic checking (Figure S1), which was consistent with an earlier study [21]. Knocking out either EPS or TasA had a minor effect on total biofilm production, but knocking out both resulted in significantly less submerged biofilm formation on cellulose (Figure 1). The wild-type strain (A_540_ = 0.624) produced 42% more biofilm than the double knockout strain (A_540_ = 0.438) (*p* < 0.001); however, no significant difference was found in the amount of biofilm produced between the wild-type strain and the Δ*tasA* strain (A_540_ = 0.579) or the Δ*eps* strain (A_540_ = 0.555) (*p* > 0.05) (Figure 1A).

### 3.2. Biofilm Is Able to Capture and Retain Cellulase

**Figure 2:**
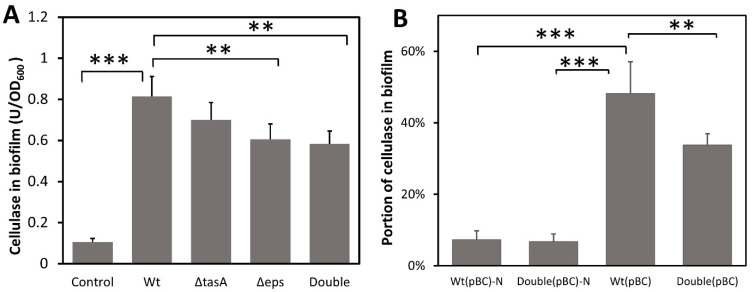
The amount of cellulase absorbed and retained by biofilm and the effect of missing biofilm matrix components. (**A**) Capture of exogenous cellulase added to the growth medium by biofilm. Cellulase was added to static cultures that promoted biofilm formation and incubated for 24 h for biofilm development. The control, to account for cellulase binding to cell surfaces, contained the same amount of added cellulase but used cells grown overnight in cultures shaken to inhibit biofilm formation. The control cells were incubated with cellulase for 2 h. Cellulase content in biofilm was normalized to cell density (OD_600_). Wt: wild-type strain; Δ*tasA*: *tasA*-knockout strain; Δ*eps: eps*-knockout strain; Double: Δ*tasA* Δ*eps* double knockout strain. (**B**) Capture of self-produced secretory cellulase by biofilm. All strains were engineered to produce a secretory cellulase. Bacteria were grown in BF medium with or without shaking for 2 days. Cellulase production can vary among strains thus the amount of cellulase in biofilm is shown as the percentage of cellulase in the biofilm relative to the total cellulase produced in the culture. Wt(pBC) and Double(pBC) are strains carrying pBC and form biofilm. Wt(pBC)-N and Double(pBC)-N are wild-type and double knockout strains, respectively, grown with shaking to inhibit biofilm formation. Data are means of cellulase content in biofilm with one standard deviation (*n* = 4–5). Statistic differences between groups were analyzed by Student’s T tests, with ** *p* < 0.01, and *** *p* < 0.001.

To test whether biofilm can capture and concentrate cellulases, *B. subtilis* strains were grown under static conditions (to promote biofilm formation) in a BF medium containing an exogenous cellulase. Because the bacterial strains we used produced little cellulase (Figure S2), consistent with a previous study [42], cellulase quantified in biofilm is a measure of cellulase sorption by the biofilm matrix. Significant amounts of exogenous cellulase were found in the biofilm produced by all strains (F_4,18_ = 61.449, *p* < 0.001) (Figure 2A). The wild-type biofilm contained 0.814 units of cellulase/OD biofilm, 1.34-fold more than that of the EPS knockout strain (0.606 U cellulase/OD biofilm) (*p* < 0.01) and 1.39-fold more than the biofilm of the double knockout strain (0.584U cellulase/OD biofilm) (*p* < 0.01), but not significantly more than that of the TasA knockout strain. To ascertain that cellulase was captured by biofilm and not simply adhering to the bacterial cell surface, a control was included whereby the wild-type strain grown to high cell density with vigorous shaking to inhibit biofilm formation was incubated with cellulase for 2 h before significant biofilm formation. Only 0.105 unit of cellulase/OD was found in cell pellets (Figure 2A). Our results suggest that EPS play a role in the retention of cellulase in biofilm and missing TasA protein has little effect on cellulase sorption. Note that the biofilm of the double knockout strain still contained more cellulase than that of the control, suggesting the presence of biofilm components other than just EPS and TasA that are involved in cellulase retention. Together, our results show that *B. subtilis* biofilm can capture cellulase from the liquid medium even when bacteria themselves did not produce the cellulase.

To confirm that *B. subtilis* biofilm can also retain self-produced cellulase, we transformed the bacterium with a cellulase over-expression plasmid, pBC, to enable their production of a secretory cellulase (Figure S2) and then measured the amount of cellulase in the biofilm. Wild-type and knockout strains with pBC produced similar amounts of cellulase when grown with vigorous shaking (Figure S2) where no biofilm is formed. As expected, the majority of the cellulase produced remained in the supernatant when cells were grown with shaking. Only 7.3% and 6.7% of the total cellulase were found in the cellular pellet for the wild-type and double knockout strains, respectively (Figure 2B). However, when cells were grown without shaking and biofilm was produced, there was a more than 6-fold increase in the proportion of cellulase retained in the wild-type biofilm (48.2%) (*p* < 0.001). The wild-type biofilm retained 1.43-fold more cellulase compared to the biofilm of the double knockout strain (33.8%) (*p* < 0.01) (Figure 2B), indicating that biofilm and particularly the EPS and TasA components facilitate retention of the secreted cellulase. The double knockout strain, despite lacking EPS and TasA, still retained some of the secreted cellulase, suggesting the presence of other biofilm components that also play a role in cellulase retention.

### 3.3. Biofilm Concentrates Exogenous Cellulase on Cellulose and Enhances Its Degradation

**Figure 3:**
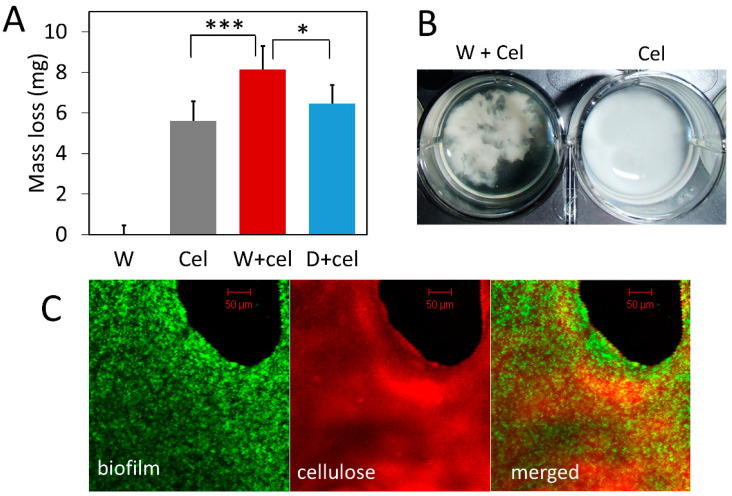
Degradation of regenerated amorphous cellulose (RAC) by exogenous cellulase with and without biofilm. W: wild-type bacteria alone; Cel: cellulase alone; W+cel: combination of wild-type bacteria and cellulase; D+cel: combination of double-knockout strain and cellulase. (**A**) Amount of RAC degraded by different treatments. Gray bars are means of mass loss with one SD (*n* = 6). (**B**) Appearance of cellulose particles in the presence and absence of bacteria. (**C**) Confocal laser scanning microscopy image of biofilm and cellulose. Wild-type bacteria labeled with green fluorescent protein with biofilm (biofilm) and RAC stained with Congo red dye (cellulose). Statistic differences between groups were analyzed by Student’s T tests, with * *p* <0.05, and *** *p* < 0.001.

Given that bacterial biofilm can capture cellulase, we speculated that localizing cellulase in biofilm close to bacteria and cellulose could facilitate cellulose degradation. To test the functional significance of biofilm in cellulose degradation, we compared cellulose degradation by exogenous cellulase alone, bacteria alone, and a combination of the two. Results show that the presence of biofilm leads to significantly greater cellulose degradation compared to the degradation by cellulase alone (Figure 3). The combination of cellulase and wild-type bacteria (W+cel) degraded 8.14 mg of cellulose in each well, which was 1.45-fold more than the amount of cellulose degraded by cellulase alone (Cel) (5.60 mg RAC/well) (*p* < 0.001) and 1.26-fold more than the amount degraded by the combination of cellulase and double-knockout strain (D+cel) (6.47 mg RAC/well) (*p* < 0.05) (Figure 3A). However, the combination of cellulase and the double-knockout strain (D+cel) did not lead to significantly more degradation of cellulose than did the cellulase alone (*p* > 0.05). It should be noted that bacterial strains used in this experiment produced no cellulase because they did not carry the pBC plasmid and alone they could not degrade any cellulose (Figure 3A). It seems likely that the increased cellulose degradation resulted from cellulase captured by biofilm and the closer proximity of captured cellulase to cellulose.

Interestingly, we noticed that cellulose particles clumped together in the presence of bacterial cells and looked different from cellulose treated with cellulase alone where cellulose particles were dispersed more homogeneously (Figure 3B). Confocal laser scanning microscopy (Figure 3C) showed that wild-type bacteria expressing GFP (green) grew densely and formed biofilm on the surface of cellulose (red). Although more sophisticated labeling of biofilm and cellulase is desirable in future studies, our result nevertheless suggests that bacterial cells, cellulases, and cellulose might form complexes with biofilm and such bacteria-cellulase-cellulose complexes could synergistically enhance cellulose degradation.

### 3.4. Biofilm Concentrates Endogenous Cellulase and Enhances Cellulose Degradation

**Figure 4:**
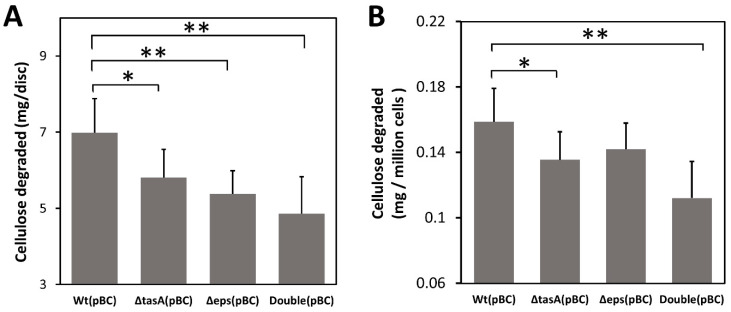
Effect missing biofilm components on cellulose degradation. (**A**) Cellulose degradation by engineered *B. subtilis* strains carrying pBC as measured by mass loss of pretreated filter paper discs. (**B**) Cellulose degradation measured by mass loss of pretreated filter paper normalized to adjust for differences in cell density. Wt: wild-type strain; Δ*tasA*: *tasA* knockout strain; Δ*eps: eps* knockout strain; Double: Δ*tasA* Δ*eps* double knockout strain. Gray bars are mean values of at least six replicates with one standard deviation. Statistic differences between groups were analyzed by Student’s T tests, with * *p* < 0.05, and ** *p* < 0.01.

To further confirm that biofilm can facilitate cellulose degradation by trapping cellulases, we next tested whether biofilm can also enhance cellulose degradation by concentrating cellulase produced by bacteria themselves without exogenous cellulase. Because the original *B. subtilis* strains we used produce little cellulase (Figure S2), we transformed all strains with a plasmid pBC overexpressing the recombinant secretory BsCel5 cellulase, previously shown to increase degrading efficiency of insoluble cellulose by *B. subtilis* [40,42]. Using bacteria producing cellulase, we found that the wild-type (pBC) strain degraded 43% more cellulose than the double knockout (pBC) strain (7.0 mg/disc versus 4.9 mg/disc) (*p* < 0.01), 30% more than the Δ*eps* (pBC) strain (5.4 mg/disc) (*p* < 0.01), and 21% more than the Δ*tas*A strain (5.8 mg/disc) (*p* < 0.05) (Figure 4A).

Differences in cellulose degradation among strains were likely not due to differential cell growth. To account for differences in cell density on filter paper discs among strains, we normalized cellulose degradation by the average number of bacterial cells (CFU) attached to each filter paper disc. The results show that the wild-type cells still degraded more cellulose than the double knockout strain after adjusting for cell density (Figure 4B). The wild-type (pBC) cells on average degraded 0.16 mg /10^6^ CFU, 45% more than the double knockout (pBC) strain (0.11 mg/million CFU) (*p* < 0.01), and 14% more than the Δ*tasA*(pBC) cells (0.14 mg/million CFU) (*p* < 0.05), but not significantly more than the Δ*eps*(pBC) cells.

## 4. Discussion

Microscopic visualization of the biofilm associated with cellulosic substrate showed positive associations between biofilm formation and cellulose degradation [6,7,8,9], but these earlier studies provide limited information about molecular mechanisms [13]. For example, *Caldicellulosiruptor obsidiansis* was found to form biofilm complexes on cellulose and these complexes were responsible for the majority of cellulose degradation; however, how biofilm interacts with cellulase and bacteria is missing [7]. With a different mechanism, *Clostridium thermocellum* produces cell-bound cellulosomes that aid the formation of a monolayer biofilm on cellulose without a polymeric matrix [9]. Until more recently, studies have found that biofilm formation on cellulose can enhance the expression of carbohydrate-active enzymes by some cellulolytic bacteria [50] and fungi [51]. In the present study, we posited that cellulose degradation might rely largely on microbes that form biofilm attached to the surface of the insoluble substrate. To obtain mechanistic details, we used the well-characterized biofilm-producing *B. subtilis* to explore how biofilm may enhance cellulose degradation, particularly the significance of EPS and TasA components in biofilm. Our results show that biofilm can concentrate free cellulases from the medium (Figure 2) and hold the enzyme together with cells and cellulose to form bacteria-cellulase-cellulose complexes that enhance cellulose degradation (Figure 3 and Figure 4). There is no evidence to suggest that cellulase retention in biofilm is enzyme-specific. Instead, the binding of macromolecules may be a general property of biofilm that allows bacteria to better utilize available resources in the surrounding medium [1,52]. The non-specific enzyme-binding capacity of biofilm may explain why different enzymatic activities have been detected in the biofilm matrix [52,53].

### 4.1. Potential Roles of Biofilm in Cellulose Degradation

Multiple interacting mechanisms may contribute to the enhanced cellulose degradation by biofilm (Figure 3 and Figure 4). Even though cellulases by themselves can bind to cellulose via their cellulose-binding domains [54], biofilm can concentrate free cellulases near bacterial cells and the substrate (Figure 2 and Figure 3). The retention of cellulase by biofilm enables the formation of bacteria-cellulase-cellulose complexes (Figure 3) that could enhance the efficiency of cellulases in cellulose degradation [54,55,56]. In these previous studies, such complexes were formed mainly through cell-bound cellulosomes that bind cellulose and bacteria together but we found that biofilm can also facilitate the formation of these complexes. Biofilm plays dual functions in promoting cellulose degradation. First, by holding bacteria close to the substrate, biofilm facilitates bacterial uptake of cellulose hydrolysis products such as glucose, and thus could increase bacterial growth and production of cellulases [9,11]. Second, the consumption of hydrolysis products by bacteria reduces product inhibition against cellulase [9,56]. Cellulase activity is inhibited by high concentrations of hydrolysis products such as oligosaccharides, cellobiose, and glucose [11,57]. In bacteria-enzyme-cellulose complexes, bacteria can effectively consume sugars released and diminish product inhibition against cellulase activity [54,55].

The exact mechanism by which biofilm captures cellulase is unknown and there is no evidence that it is specific. Since no cellulose biosynthetic gene cluster has been identified in *B. subtilis* [15,16] and thus cellulose cannot be produced in the biofilm matrix of this bacterium, it is unlikely that cellulase is captured via its specific binding to cellulose in the biofilm matrix. Possible mechanisms for cellulase capture by biofilm include encapsulation by extracellular polymeric substances, electric attraction by charged polysaccharides and proteins, and hydrophobic interactions with lipids, lipopolysaccharides, and lipopeptides [1,58]. *B. subtilis* produces a complex biofilm matrix and one or more of the mechanisms may be involved. For example, because TasA and EPS can assemble the biofilm matrix extracellularly [21], free cellulases and other extracellular enzymes may be captured and integrated into the biofilm matrix during the assembly process.

### 4.2. Biofilm Resolves the Dilemma of Bacteria Producing Free Cellulases in the Environment

Cellulose degradation benefits bacteria by providing carbon sources but it is a challenge because the hydrolysis products are released into the environment [7,9,11]. Some cellulolytic bacteria such as *Clostridium thermocellum* are able to produce cell-bound cellulosomes that can bind to cellulose via its cellulose-binding proteins [56,59]. Cellulosomes enable the formation of bacteria-cellulase-cellulose complexes where host bacteria can directly absorb hydrolysis products and thus have an advantage over competing bacteria [11,59]. However, bacteria such as *B. subtilis* that secrete free or non-cell-bound cellulases have a dilemma. Cellulase must be produced to digest cellulose but the hydrolysis products can be absorbed by surrounding competitors if the cellulases are released into the environment. This dilemma is resolved by producing a biofilm capable of retaining secreted cellulases as shown in the present study. Biofilm improves the cellulose degradation efficiency by concentrating active enzymes on the substrate, promoting synergy between bacteria and cellulases as discussed above.

One potential limitation of the study was that we could not completely abolish submerged biofilm formation on cellulose even though we deleted the genes for two major biofilm components EPS and TasA. Biofilm formation in *B. subtilis* involves many complicated genetic factors including some unknown. Improved understanding of how biofilm facilitates cellulose degradation would benefit from the identification of other biofilm components in future studies. We also considered the possibility of mutation off-target effects but this does not seem likely because all mutants were made by the in-frame replacement method which has shown little polar effects on downstream genes [27] and which was also used to create Δ*tasA* and Δ*eps* mutants in previous studies [21,28].

In this work, we mainly focused on the biofilm of a single species, *B. subtilis*. It is well-known that many other bacteria can also form robust biofilm and it would be interesting to know whether biofilm from other bacteria plays similar roles in cellulose degradation in the environment as cellulose is highly abundant in nature and serves as a carbon source during bacterial growth. In addition, different bacteria could also form mixed-species biofilm where they contribute different biofilm components and cellulases. Perhaps mixed-species biofilm could degrade cellulose faster than mono-species biofilm as cellulose degradation was found to be a synergistic process among different bacterial species [60].

Due to its potential applications, biofilm has received increasing attention for its effects on lignocellulose degradation and conversion [13,61]. For example, consolidated bioprocessing (CBP), which relies on biofilm formation, offers a promising and economic solution to produce useful chemicals and biofuels from lignocellulose [11,13,61]. Our finding that EPS and TasA protein can capture free cellulases suggests the possibility of genetic manipulation of these components or others to make biofilms more effective in enzyme capture/localization for efficient cellulose degradation and conversion. Biofilm matrix could also be engineered to facilitate the formation of bacteria-enzymes-cellulose complexes that degrade cellulose more efficiently.

In conclusion, our results show that biofilm formation by *B. subtilis* facilitates cellulose degradation. Bacillus biofilm can retain and capture free cellulases from the surrounding medium, allowing the formation of bacteria-enzyme-cellulose complexes. The formation of such complexes facilitates the synergistic degradation of cellulose by *B. subtilis*. Given the ubiquitous presence of biofilm in nature, it is likely that biofilm from other bacteria also aids in the decomposition of cellulose and other complex organic matters. A deeper understanding of the functions of biofilm components and how they interact with cellulases can also be useful to the future development of biofilm-related biotechnologies as a new way to improve biomass degradation for cost-effective biofuel production.

## Figures and Tables

**Table 1:** *Bacillus subtilis* strains and plasmids used in the study.

Strain (Plasmid)	Relevant Genotype or Property	Source
Wt	Wild type 168, *trpC2*	BGSC *
Δ*eps*	Δ*eps*::*zeoR*; *eps* operon deleted from Wt168	This study
Δ*tasA*	Δ*tasA*::*ermR*; *tasA* deleted from Wt168	BGSC
Double	Δ*eps*::*zeoR*, Δ*tasA*::*ermR*; Δ*eps* Δ*tasA* double knockout from Wt 168	This study
*E. coli* DH5α (p7Z6)	*ampR*, *zeoR*, *lox71-zeo-lox66*	[30]
*B. subtilis* 1012M15 (pGDV1)	High-copy-number plasmid, camR; carrying multiple cloning site mp18	[32]
*E. coli* DH5α (pDR111-GFP(Sp))	ampR, specR, carrying GFP; integration vector for *B. subtilis*	[33]
Wt(pBC)	Wt168 carrying cellulase over-expression plasmid pBC; *camR*	This study
Δ*eps*(pBC)	Δ*eps* carrying pBC	This study
Δ*tasA*(pBC)	Δ*tasA* carrying pBC	This study
Double(pBC)	Δ*eps* Δ*tasA* carrying pBC	This study
Wt_GFP	Wt168 with GFP incorporated into its genome by plasmid pDR111-GFP(Sp)	This Study

* BGSC: Bacillus Genetic Stock Center. *camR*, chloramphenicol resistance gene; *ampR*, ampicillin resistance gene; *specR*, spectinomycin resistance gene; *zeoR*, zeocin resistance gene; *ermR*, erythromycin resistance gene.

**Table 2:** Primers used in the study. Bold and underlined: sequences that overlap with *zeoR* used to create the *eps* knockout cassette.

Primer Name	Primer Sequence 5′-----3′
eps-up-F	ACGCCATTGTCCGACAGG
eps-up-R	**AATCTCTAGAGGATCCCCGGGTACCGAGCTC** CTCATTCATGTATTCATAGCCTTCAGC
zeo-F	GAGCTCGGTACCCGGGGATCCT
zeo-R	GCTTGCATGCCTGCAGGTCGAC
eps-dw-F	**GTAGAATCGTCGACCTGCAGGCATGCAAGC**ATCCAGCACGCCTCA AAGAAG
eps-dw-R	TATCTTGAATGGTATGAAGCGGAAT
epsCF-F	GACTGAGCAGGTTCCAATC
epsCF-R	CCAAGTTGAGCGAGTGTTTC
tasA-F	CATCAGGTACGCTTGATTTATCTG
tasA-R	GTTTTATCATCCTTGAATTGGATTTCC

## Data Availability

The datasets generated for this study are available on request to the corresponding author.

## Supplementary Materials

The following supporting information can be downloaded at: https://www.mdpi.com/article/10.3390/microorganisms10081505/s1, Figure S1: Characterization of biofilm knockout strains of *B. subtilis*; Figure S2: Cellulase production by *B. subtilis* strains in BF medium with vigorous shaking.
