# Supplementary material for: Sorption of Cellulases in Biofilm Enhances Cellulose Degradation by Bacillus subtilis
Source: Microorganisms. 2022 Jul 26;10(8):1505. doi: 10.3390/microorganisms10081505 (PMC9329931; doi:10.3390/microorganisms10081505)
Supplement: Supplementary file 1 [file microorganisms-10-01505-s001.zip › microorganisms-1808476-supplementary.pdf]

**Supplementary Material**

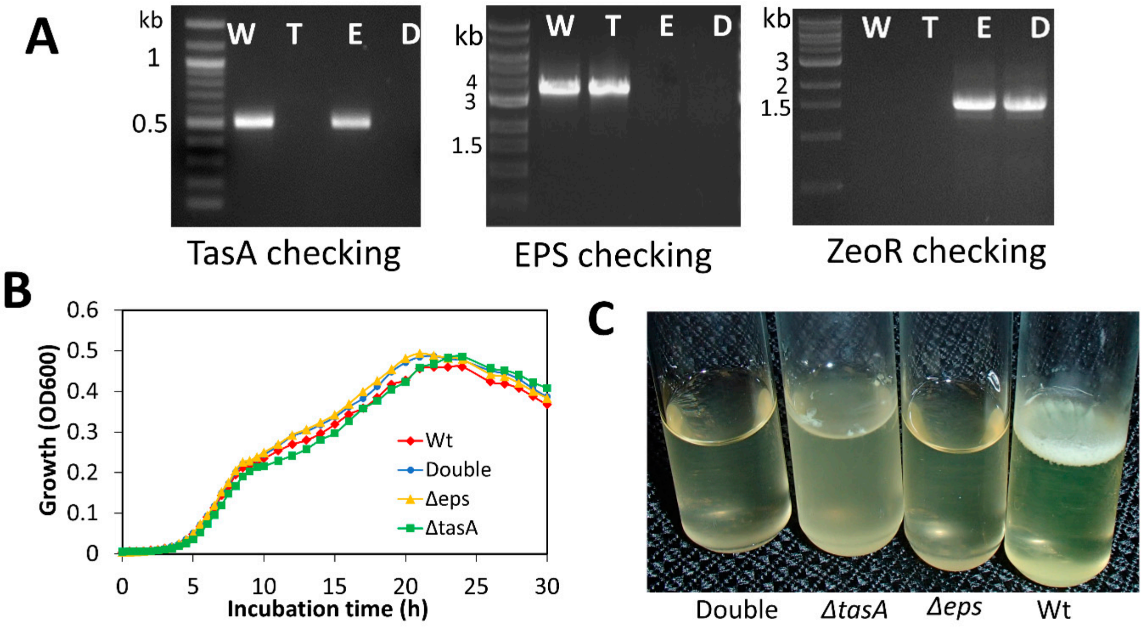


**Figure S1**. Characterization of biofilm knockout strains of B. subtilis. (A) Verification of gene deletion and presence of ZeoR insert by PCR with gene-specific primers. W: wild-type strain; T: ΔtasA knockout strain; E: Δeps knockout strain; D: double-knockout (*Δeps ΔtasA*) strain. The Δ presence of *tasA* gene was checked using the primer set tasA-F and tasA-R and the expected product size is ~0.5 kb. The presence of *eps* operon was checked using primer set epsCF-F and epsCF-R and the expected product size is ~3.5 kb. The presence of ZeoR insert was checked using primer set eps-up-F and Zeo-R and the expected product size is ~1.5kb. (B) Growth of all strains under vigorous shaking condition. Values are means of three replicates. The standard deviation of each data point was within 15% of the mean and thus not shown. (C) The effect of gene deletion on pellicle biofilm formation by *B. subtilis* strains. Double: *ΔtasA Δeps* double knockout strain; *Δeps*: *eps* knockout strain; *ΔtasA*: *tasA* knockout strain; Wt: wild-type strain. Bacteria (initial OD =0.01) were first grown in glass test tubes (18 mm × 150 mm) containing BF medium (8 ml) with shaking at 200 rpm at 30°C for 6 h and then without shaking for 3 days.

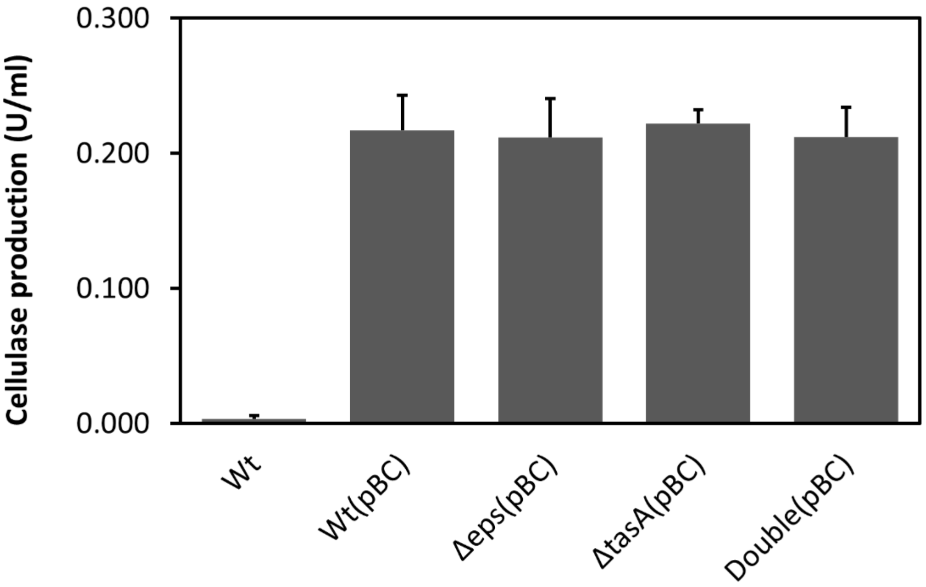


**Figure S2**. Cellulase production by *B. subtilis* strains in BF medium with vigorous shaking. Wt: wild-type strain without pBC plasmid; Wt(pBC): wild-type strain with pBC plasmid; Δeps(pBC): *eps* knockout strain with pBC plasmid; *ΔtasA*(pBC): *tasA* knockout strain with pBC plasmid; Double(pBC): double-knockout strain with pBC plasmid. There was no significant difference in cellulase production among bacterial strains with the pBC plasmid ($F_{3,12}$ =0.1795, $P = 0.908$). Bacteria grew similarly and were normalized to the same density. Each bar represents the mean of four replicates with one standard deviation.
